# Silencing of PINK1 Expression Affects Mitochondrial DNA and Oxidative Phosphorylation in DOPAMINERGIC Cells

**DOI:** 10.1371/journal.pone.0004756

**Published:** 2009-03-09

**Authors:** Matthew E. Gegg, J. Mark Cooper, Anthony H. V. Schapira, Jan-Willem Taanman

**Affiliations:** Department of Clinical Neurosciences, Institute of Neurology, University College London, Queen Square, London, United Kingdom; Buck Institute for Age Research, United States of America

## Abstract

**Background:**

Mitochondrial dysfunction has been implicated in the pathogenesis of Parkinson's disease (PD). Impairment of the mitochondrial electron transport chain (ETC) and an increased frequency in deletions of mitochondrial DNA (mtDNA), which encodes some of the subunits of the ETC, have been reported in the substantia nigra of PD brains. The identification of mutations in the *PINK1* gene, which cause an autosomal recessive form of PD, has supported mitochondrial involvement in PD. The PINK1 protein is a serine/threonine kinase localized in mitochondria and the cytosol. Its precise function is unknown, but it is involved in neuroprotection against a variety of stress signalling pathways.

**Methodology/Principal Findings:**

In this report we have investigated the effect of silencing PINK1 expression in human dopaminergic SH-SY5Y cells by siRNA on mtDNA synthesis and ETC function. Loss of PINK1 expression resulted in a decrease in mtDNA levels and mtDNA synthesis. We also report a concomitant loss of mitochondrial membrane potential and decreased mitochondrial ATP synthesis, with the activity of complex IV of the ETC most affected. This mitochondrial dysfunction resulted in increased markers of oxidative stress under basal conditions and increased cell death following treatment with the free radical generator paraquat.

**Conclusions:**

This report highlights a novel function of PINK1 in mitochondrial biogenesis and a role in maintaining mitochondrial ETC activity. Dysfunction of both has been implicated in sporadic forms of PD suggesting that these may be key pathways in the development of the disease.

## Introduction

Parkinson's disease (PD) is characterized by loss of dopaminergic neurons in the substantia nigra of the brain, with surviving neurons typically containing intracytoplasmic protein inclusions known as Lewy bodies [1]. Decreased activity of complex I of the electron transport chain (ETC) has been reported in PD substantia nigra [2], while toxins that inhibit complex I, such as 1-methyl-4-phenyl-1,2,3,6-tetrahydropyridine and rotenone, can induce parkinsonian features in humans and animal models [1], [3]. Furthermore, deleted mitochondrial DNA (mtDNA) species have been reported to accumulate in the neurons of PD substantia nigra [4], while mice with reduced mtDNA copy number in dopaminergic neurons exhibit a parkinsonian phenotype [5].

Genes associated with familial forms of PD have also been shown to effect mitochondrial function [1], [6]. In particular, the identification of mutations in *PINK1* has strongly implicated mitochondrial dysfunction in the pathogenesis of PD [7]. The PINK1 protein is a serine/threonine kinase that has been localized to the cytosol and the inner membrane of mitochondria [8], [9]. PINK1 is capable of autophosphorylation [10], [11] and has been shown to be involved in the phosphorylation of the mitochondrial chaperone TRAP1 [12] and the mitochondrial protease HtrA2 [13]. Disruption of PINK1-mediated phosphorylation of either TRAP1 or HtrA2 resulted in increased cell death. Over-expression of wild type PINK1, but not mutant PINK1, also prevents loss of mitochondrial membrane potential and increased release of cytochrome-*c* from mitochondria following stress [7], [14], [15].

A PINK1 knockout mouse model has linked PINK1 with a role in dopamine release and striatal plasticity [16], as well as defects in mitochondrial respiration [17]. In addition, abnormal mitochondrial morphology has been observed in the flight muscles of *Drosophila* lacking PINK1, and in human cells either harboring PD-associated PINK1 mutations or with reduced PINK1 expression [18]–[23]. In the *Drosophila* models, loss of PINK1 function resulted in mitochondrial dysfunction in flight muscle and loss of dopaminergic neurons [19], [20]. These *Drosophila* models have also shown that parkin, another protein associated with familial forms of PD, acts downstream of PINK1 in a putative common pathway [18]–[21].

The dysfunction of mitochondria described above, coupled with perturbed ETC activity in sporadic PD, prompted us to investigate the role of PINK1 on oxidative phosphorylation and mitochondrial biogenesis in the human dopaminergic SH-SY5Y neuroblastoma cell line.

## Materials and Methods

### Cell culture

The human SH-SY5Y neuroblastoma cell line was cultured in 1∶1 (v/v) DMEM∶F12 (Ham) media containing 0.9 g/l glucose and supplemented with 10% fetal bovine serum, 1 mM sodium pyruvate, and penicillin-streptomycin.

For the generation of stable cell lines over-expressing PINK1 or parkin, SH-SY5Y cells were transfected with pCMV6-Neo vector (Origene) containing full-length wild type PINK1 cDNA, or pcDNA3.1 vector (Invitrogen) containing parkin cDNA with a HA epitope added to the C-terminus. Stable cell lines were selected by supplementing the media with 40 µg/ml G418. DNA extracted from both cell lines was sequenced to confirm cDNA sequences. Over-expression of the respective recombinant proteins was verified by western blot with a PINK1 antibody (recombinant PINK1 protein has no epitope tags), or antibodies specific for parkin or the C-terminus HA epitope tag.

### Transient transfection of SH-SY5Y cells with PINK1 siRNA

SH-SY5Y cells (1.8×10^5^ cells/ml) were transfected with a pair of PINK1 siRNAs (5 nM each) or 10 nM scrambled control siRNA (Ambion negative control siRNA #1) using HiPerfect transfection reagent (Qiagen). Two combinations of PINK1 siRNA were tested, with each siRNA targeting a different region of PINK1 mRNA. Pair 1 siRNAs (PINK1 #1; sense strands: GGACGCUGUUCCUCGUUAU and AAGCCACCAUGCCUACAUUUU) were designed and synthesized by Dharmacon. Pair 2 siRNAs (PINK1 #2) were Qiagen HP validated siRNAs (S100287931, sense strand: GACGCUGUUCCUCGUUAUGAA and S100287924, sense strand: CCGGACGCUGUUCCUCGUUAU). Cells were passaged and transfected with siRNA every 3 days when the cells were 80–90% confluent and in log growth phase. The transfection efficiency of FAM-labeled negative control siRNA (Ambion) was >90% after both the first and last transfection. For SH-SY5Y cells over-expressing recombinant PINK1, cells were transfected with either 50 nM of the PINK1 siRNA pairs or scrambled control siRNA for 40 hours.

### Quantitative real-time PCR of mRNA and mtDNA

RNA was extracted from siRNA-treated cells using the RNeasy kit (Qiagen) and converted to cDNA by the QuantiTect reverse transcription kit (Qiagen). mRNA levels were measured by quantitative real-time PCR (qPCR) using the QuantiTect SYBR Green kit (Qiagen). For primer sequences see Table S1. GAPDH was amplified as the reference mRNA. Relative expression was calculated using the ΔC_T_ method.

For measurement of mtDNA, total DNA was extracted from siRNA-treated cells using the QIAamp DNA mini-kit (Qiagen). MtDNA levels were measured by quantitative real-time PCR using primers in the D-loop of mitochondria (Table S1) [24]. Amplification of the D-loop of mitochondria was measured using the QuantiTect SYBR Green kit, and was expressed relative to the single copy nuclear gene *TK2*. Relative expression was calculated using the ΔC_T_ method.

### Radioactive mtDNA synthesis assay

MtDNA synthesis was measured in cells by measuring the incorporation of [methyl-^3^H]thymidine into mtDNA. SH-SY5Y cells previously treated with siRNA for 72 hours were passaged into a 24-well plate, transfected with siRNA once more, and incubated at 37°C for 72 hours. For the last 18 hours, cells were treated with 10 µg/ml aphidicolin and 5 µCi/well [methyl-^3^H]thymidine (GE Healthcare). Cells were washed three times with phosphate-buffered saline and lysed overnight in 0.25 M NaOH. A fraction of lysed cells was then spotted onto DE81 anion exchange paper (Whatman) and washed twice with 300 mM sodium chloride, 30 mM sodium citrate buffer (pH 7.0) and once with ethanol to remove unincorporated [methyl-^3^H]thymidine. After drying, the anion exchange paper was placed in scintillant and radioactivity measured. Each sample was measured in triplicate. Immunodetection of 5-bromo-2′-deoxyuridine incorporation indicated that nuclear DNA replication was drastically inhibited by aphidicolin (Sigma-Aldrich). To allow for nuclear DNA incorporation that was insensitive to aphidicolin treatment, [methyl-^3^H]thymidine was measured in SH-SY5Y cells lacking mtDNA (rho zero cells). Incorporation was typically reduced by >95% in these cells, and was subtracted from the values obtained for siRNA-treated SH-SY5Y. Data were expressed as ^3^H counts per minute incorporated into DNA/mg of protein over an 18-hour period.

### Western blotting

For detection of PINK1 expression, SH-SY5Y cells treated with siRNA were harvested and lysed on ice with 0.25% (v/v) Triton X-100 in phosphate-buffered saline, supplemented with protease inhibitors (1 mM phenylmethanesulfonyl fluoride, 1 µg/ml of pepstatin A, 1 µg/ml of leupeptin) and 1 mM EDTA for 30 minutes. The soluble fraction (10 µg) was resolved by SDS-PAGE and transferred to Hybond P (GE Healthcare). Blots were probed with PINK1 antibody (clone BC100-494, Novus Biologicals) and bands detected by enhanced chemiluminescence (Pierce). Equal loading was assessed using an antibody against succinate dehydrogenase subunit SDHA of the electron transport chain (clone 2E3, Mitosciences).

For the detection of subunit MTCO2 of complex IV (clone 12C4, Mitosciences), porin (clone 89-173/016, Calbiochem) and TFAM (clone K-18, Santa-Cruz Biotechnology), siRNA-treated cells were lysed with 1.5% (v/v) n-dodecyl-β-D-maltoside (Anatrace) in phosphate-buffered saline, supplemented with protease inhibitors, and processed as above. Equal protein loading was assessed using a GAPDH antibody (clone 6C5, AbCam). Density of bands was quantified by AlphaDigiDoc software.

Preparation of blue native gels and subsequent western blotting is detailed in supplementary Method S1.

### Measurement of mitochondrial enzyme activities

Following treatment with siRNA, cells were harvested and resuspended in isolation medium (320 mM sucrose, 10 mM Tris, 1 mM EDTA, pH 7.4) and activity measured by spectrophotometric methods as previously described [25], [26].

### Measurement of cellular steady state ATP levels

Following treatment of SH-SY5Y cells with siRNA for 12 days, cells were trypsinized and resuspended in phosphate-buffered saline (1×10^5^ cells/ml). ATP levels were measured using the ATP Bioluminesence Assay kit HSII (Roche). Each sample was measured in triplicate. For the oligomycin experiments, cells were treated with 1 µg/ml of oligomycin for 30 minutes prior to harvesting cells.

### ATP synthesis assay

SH-SY5Y cells were treated with siRNA for 12 days, trypsinized and washed three times with ice cold phosphate-buffered saline. Cells were resuspended at 2×10^5^ cells/ml in incubation medium (25 mM Tris, 150 mM KCl, 2 mM K^+^-EDTA, 10 mM K_2_HPO_4_, pH 7.4). An aliquot of cells (2×10^4^) was mixed with an equal volume of incubation buffer containing 1 mg/ml of bovine serum albumin, 1 mM ADP and substrates (complexes I, III, IV: glutamate+malate (10 µM); complexes II, III, IV: succinate (10 µM)+rotenone (40 µg/ml); complex IV: ascorbate (2 mM)+*N*,*N*,*N′*,*N′*-tetramethyl-p-phenylenediamine (50 µM)), permeabilized with digitonin (40 µg/ml), and incubated at 37°C for 20 minutes. The reaction was stopped with perchloric acid, and samples neutralized with 3 M K_2_CO_3_ dissolved in 0.5 M tri-ethanolamine. Debris was removed by centrifugation and ATP measured with the ATP Bioluminesence Assay kit HSII. Data were expressed as pmoles ATP synthesized/minute/10^5^ cells. ATP synthesis was virtually abolished when the assays were performed with an appropriate inhibitor (e.g. glutamate+malate in the presence of rotenone). Furthermore, ATP synthesized in cells in the absence of substrates was ≤2%, when compared to cells with substrates.

### Assessment of mitochondrial membrane potential

Mitochondrial membrane potential was assessed in SH-SY5Y cells with the probe JC-1 (Invitrogen). Cells were treated with siRNA for 12 days in 24-well plates and loaded with 3 µg/ml of JC-1 for 30 minutes at 37°C. The cells were washed with phosphate-buffered saline, and mitochondrial JC-1 aggregates were measured with a fluorescent plate reader (excitation 530 nm, emission 590 nm). In a sister well, JC-1 fluorescence was measured in the presence of the ionophore valinomycin (100 nM), which destroys the mitochondrial membrane potential, and was subtracted from the data. JC-1 fluorescence was expressed against protein.

### Glutathione quantification

Cells treated with siRNA were harvested and the pellet resuspended in isolation medium. Reduced glutathione (GSH) levels were determined electrochemically following extraction into 15 mM ortho-phosphoric acid and separation by reverse-phase high performance liquid chromatography [27].

### Detection of protein carbonyls

Protein carbonyl levels were measured in SH-SY5Y treated with siRNA for 6 or 12 days using the Oxyblot protein oxidation detection kit (Millipore). Briefly, cells were lysed with 0.25% (v/v) Triton X-100 in phosphate-buffered saline supplemented with 50 mM DTT and protease inhibitors. Following removal of insoluble material, protein carbonyls were derivatized to 2,4-dinitrophenylhydrazone (DNP) by reaction with 2,4-dinitrophenylhydrazine. The DNP-derivatized protein samples were then separated by SDS-PAGE followed by western blotting. Carbonyls were detected by incubation with an antibody specific to DNP. Note that protein samples which were not derivatized to DNP were not detected by the DNP antibody. Equal loading was determined by reprobing the same blot with GAPDH antibody.

### MitoTracker Green staining of SH-SY5Y cells

The mitochondrial network in live SH-SY5Y treated with siRNA for 12 days was assessed with MitoTracker Green FM (Invitrogen). Cells were cultured on coverslips or in 24 well culture plates and incubated with 5 µg/ml 4′-6-diamidino-2-phenylindole (DAPI) for 2 hours in culture media. The cells were then loaded with 250 nM MitoTracker Green FM in culture media for 45 minutes at 37°C. Cells were washed once in phenol red free media and images captured using a Zeiss Axioplan fluorescent microscope or fluorescence quantified using a fluorescent plate reader.

### Measurement of cell viability and apoptosis

Cell death was measured by the Cell Titer-Blue cell viability assay (Promega). For the Cell Titer-Blue assay, cells were treated with or without 0.5 mM paraquat (Aldrich) for 24 hours. For the last 4 hours, Cell Titer-Blue was added to each well, and fluorescence measured (excitation 530 nm, emission 590 nm). The mean fluorescence of three wells was calculated and expressed as the percentage fluorescence of untreated cells in sister wells.

For the detection of active caspase-3 and cytochrome-*c* release from mitochondria, 12-day siRNA-treated cells were grown on coverslips and treated with 0.5 mM paraquat for the last 24 hours. Cells were fixed with 3.7% paraformaldehyde, permeabilized with methanol (−20°C) and incubated with anti-cytochrome-*c* (clone 6H2.B4, BD Biosciences) and anti-active caspase-3 (clone 5A1, Cell Signaling). Cytochrome-*c* and active caspase-3 were detected using goat anti-mouse Alexa 488 and goat anti-rabbit Alexa 568 (Invitrogen), respectively. Nuclei were stained with DAPI, and the fraction of cells positive for active caspase-3 in each field of view was determined. The average of five randomly selected fields for each condition was calculated. Immunofluorescence was performed on four separate siRNA experiments.

### Statistical Analysis

Data are expressed as the mean±*s.e.m.* of separate siRNA experiments. Statistical significance between PINK1 siRNA-treated and scrambled control siRNA-treated SH-SY5Y cells was determined by one-way ANOVA and Tukey HSD test.

## Results

### Silencing of PINK1 expression

**Figure 1:**
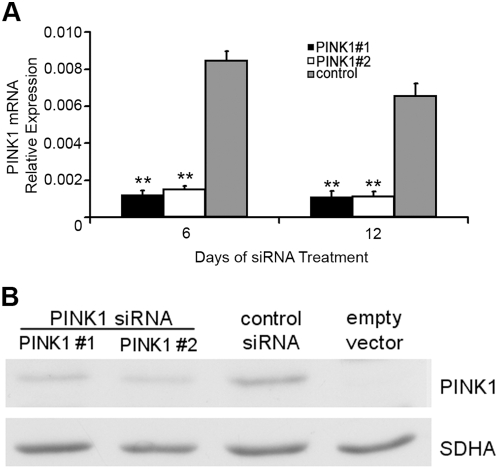
Silencing of PINK1 mRNA and protein expression in SH-SY5Y cells. A, SH-SY5Y cells were transfected with PINK1 siRNA (PINK1 #1 or PINK1 #2) or scrambled control siRNA for 6 or 12 days and PINK1 mRNA levels measured by reverse transciption and quantitative real-time PCR. Relative expression was determined against GAPDH mRNA levels and data were expressed as mean±*s.e.m.* (*n* = 4). **P<0.01 vs. scrambled control siRNA. B, SH-SY5Y cells over-expressing recombinant PINK1 protein were transfected with either PINK1 siRNA (PINK1 #1 or PINK1 #2) or scrambled control siRNA for 40 hours. The expression of full-length recombinant PINK1 protein was determined in cell lysates by western blotting. No band was detected in SH-SY5Y transfected with empty vector. Protein loading was verified with antibody against succinate dehydrogenase subunit SDHA.

Two different PINK1 siRNA pairs (PINK1 #1 and PINK1 #2) were used in all experiments in order to confirm that results are a direct consequence of PINK1 silencing, rather than an off-target effect. Transfection of SH-SY5Y cells every three days with either combination of PINK1 siRNA resulted in a sustained decrease in PINK1 mRNA levels after 6 and 12 days, with mRNA levels decreased by >80% at both time points (*P*<0.01; Figure 1A). The Titer-Blue cell viability assay indicated that silencing of PINK1 for up to 12 days had no effect on cell death under basal conditions, when compared to cells transfected with scrambled control siRNA (PINK1 #1, 65,749±4,578 fluorescent units; PINK1 #2, 58,234±3,836; control siRNA, 59,688±1,351 (mean±s.e.m., *n* = 4)).

In agreement with a previous report [28], low levels of endogenous PINK1 protein expression in SH-SY5Y was detected by the PINK1 antibody after long exposure (Figure S1). The large number of non-specific bands, combined with the small amount of protein obtained from siRNA experiments and difficulties in solubilizing PINK1 [8], [29] meant that reliable detection of PINK1 protein expression following siRNA treatment was not possible. However, over-expression of full length recombinant PINK1 (∼60 kDa) in SH-SY5Y could easily be detected by the PINK1 antibody (Figure 1B and Figure S1). Unfortunately, processed PINK1 (∼55 kDa) co-migrated with a non-specific band (Figure S1). Recombinant PINK1 mRNA levels were 10-fold higher than endogenous levels in this cell line. Treatment of these cells with the two different pairs of PINK1 siRNA reduced PINK1 mRNA levels by 60% when compared to scrambled control siRNA-treated cells. This resulted in a decrease in recombinant PINK1 protein expression, with the PINK1 #2 siRNA pair more effective (52% decrease compared to scrambled control siRNA; *n* = 3) than PINK1 #1 siRNA (39% decrease; *n* = 3) (Fig. 1B). This suggests that silencing of endogenous PINK1 mRNA levels by >80% (Figure 1A) does result in decreased endogenous protein expression.

### MtDNA levels and mtDNA synthesis

**Figure 2:**
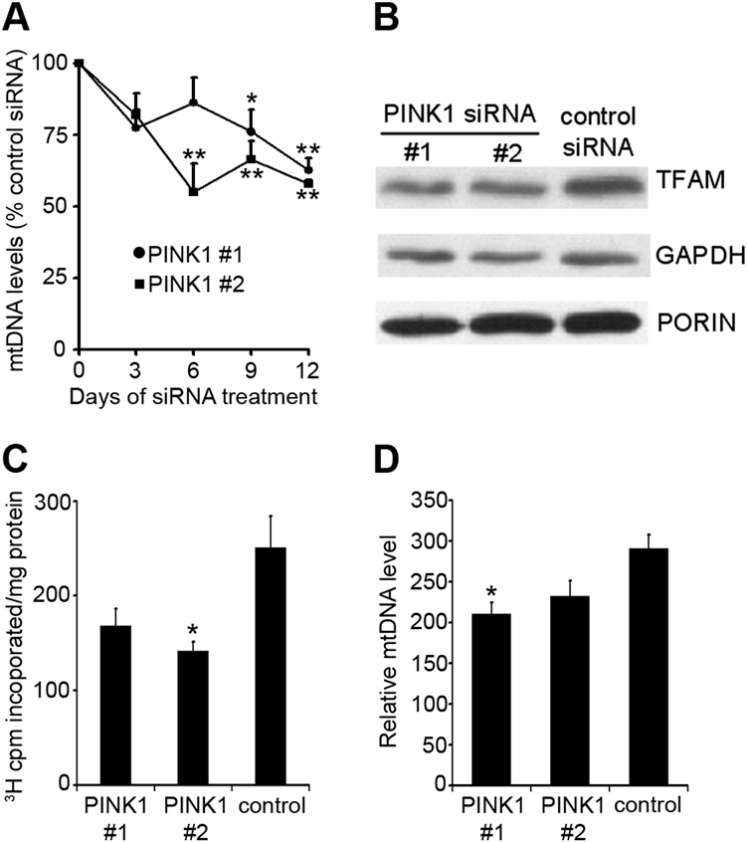
Measurement of mtDNA levels and synthesis in PINK1-silenced SH-SY5Y cells. A, SH-SY5Y cells were treated with PINK1 or scrambled control siRNA for 3, 6, 9 or 12 days and mtDNA levels measured by quantitative real-time PCR. MtDNA levels were expressed relative to the single copy nuclear gene *TK2*. Data are expressed as percentage of control siRNA mtDNA levels (*n* = 4). B, cells were treated with siRNA for 12 days and cell lysates probed for TFAM, GAPDH and porin by western blot. C, SH-SY5Y cells were treated with siRNA for 6 days and *de novo* mtDNA synthesis measured by quantifying the incorporation of [methyl-^3^H]thymidine into mtDNA over an 18-hour period in the presence of aphidicolin. Data are expressed as 10^3^ counts per minute incorporated per mg of protein and are the mean±s.e.m (*n* = 4). D, SH-SY5Y were treated with PINK1 siRNA for 6 days in the presence of 10 µM ddC, and then the ddC washed away, and the cells incubated for a further 3 days in the presence of siRNA before mtDNA levels were measured (*n* = 4). * *P*<0.05 vs. scrambled control siRNA; **P<0.01 vs. scrambled control siRNA.

MtDNA levels were measured in SH-SY5Y cells after silencing of endogenous PINK1 by quantitative real-time PCR (Figure 2A). MtDNA levels were significantly decreased with PINK1 #2 siRNA after 6 days (*P*<0.05), and were depleted by 38% (PINK1 #1) or 42% (PINK1 #2) after 12 days (*P*<0.01), when compared to control scrambled siRNA-treated cells. These results were confirmed by Southern blot, with mtDNA levels decreased by 20.5% and 35.7% in SH-SY5Y treated with PINK1 #1 and #2 siRNA, respectively, for 6 days. Western blotting for mitochondrial transcription factor A (TFAM), a protein that binds to mtDNA and whose expression closely mirrors mtDNA levels [30], also suggested that mtDNA levels were decreased in PINK1 silenced cells after 12 days (Figure 2B). TFAM protein levels were decreased by 39% (PINK1 #1) or 30% (PINK1 #2) when compared to scrambled control siRNA (*P*<0.05; *n* = 4). The decreased mtDNA levels were not due to a lower mitochondrial number. Citrate synthase activity (PINK1 #1, 817.4±71.6 nmol/min/mg protein; PINK1 #2, 799.1±95.0; scrambled control siRNA, 858.1±139.6; mean±s.e.m., *n* = 9) were unaffected by PINK1 silencing. The activity of malate dehydrogenase, another citric acid cycle enzyme, was also unaffected in PINK1-silenced cells (PINK1 #1, 13.9±1.5 µmol/min/mg protein; PINK1 #2, 17.1±2.0; scrambled control siRNA, 19.7±0.8; mean±s.e.m., *n* = 4). Furthermore, the expression of the mitochondrial protein porin was similar in both PINK1 #1 and PINK1 #2 siRNA-treated cells (105% and 99%, respectively), when normalized to GAPDH expression and compared to control siRNA-treated cells (Figure 2B). Mitochondrial mass was also determined in live cells using the specific mitochondrial probe MitoTracker Green FM. The mitochondrial fluorescence in each well was normalized to cell number by expressing against DAPI fluorescence. Once again, there was no difference in mitochondrial fluorescence between PINK1-siRNA treated and control siRNA-treated cells (PINK1 #1, 1.41±0.17; PINK1 #2, 1.56±0.23; control siRNA, 1.46±0.05 (mean±s.e.m., *n* = 4)).

We next determined whether the lower mtDNA levels in PINK1-silenced cells was due to a decrease in the rate of mtDNA synthesis by quantifying the incorporation of [methyl-^3^H]thymidine into mtDNA of siRNA-treated cells over an 18-hour period in the presence of aphidicolin, an inhibitor of nuclear DNA polymerases. Incorporation of [methyl-^3^H]thymidine into mtDNA of PINK1 #2 siRNA-treated cells was significantly inhibited by 44% (*P*<0.05; Figure 2C).

The nucleotide 2′3′-dideoxycytidine (ddC) competitively and reversibly inhibits DNA polymerase-γ (the enzyme responsible for the synthesis and repair of mtDNA) and is commonly used to deplete mtDNA levels in cultured cells [31]. In sister wells, cells were transfected with siRNA as normal, but were cultured in media containing 10 µM ddC for 6 days. After 6 days, one well was harvested and mtDNA levels measured by quantitative real-time PCR, while in the sister well, the ddC was washed away, and transfected with PINK1 or control siRNA for a further three days before mtDNA levels were measured. The removal of ddC from the cell culture media should reverse the inhibition of DNA polymerase-γ during these three days, resulting in *de novo* mtDNA synthesis. Following 6 days of siRNA and ddC treatment, mtDNA levels were depleted to a similar extent in PINK1 and control siRNA treated cells (PINK1 #1 mtDNA relative expression, 291±57; PINK1 #2, 267±41; control siRNA, 254±62). After the ddC was washed away, mtDNA levels in control siRNA treated cells increased by 13%. However, in PINK1-silenced cells, mtDNA levels continued to decrease (PINK1 #1, 29%; PINK1 #2, 13%) following the removal of ddC, and were significantly lower than control siRNA-treated cells (*P*<0.05; Figure 2D). This result indicates that that *de novo* mtDNA synthesis was reduced in PINK1-silenced cells.

Since PINK1 appears to occur upstream of parkin in a signaling pathway [18]–[22], and parkin has been implicated in mitochondrial biogenesis [32], we investigated whether over-expression of parkin could reverse the mtDNA depletion observed following PINK1-silencing. SH-SY5Y cells constitutively over expressing wild-type parkin were treated with PINK1 or control siRNA for 6 days and mtDNA relative expression measured. MtDNA levels were decreased by 28% and 40% (P<0.05) in cells treated with PINK1 #1 and PINK1#2 siRNA, respectively (Figure S2). This depletion of mtDNA following PINK1-silencing was similar to parallel experiments in which wild-type SH-SY5Y cells were treated with PINK1 siRNA (Figure S2). Therefore, this would suggest that the regulation of mtDNA by PINK1 is not mediated via parkin.

### Mitochondrial ETC activity

Given the decrease in mtDNA levels observed in PINK1-silenced cells, the activities of the ETC enzyme components were measured after 6 and 12 days of siRNA treatment, and expressed against citrate synthase activity. After 6 days of PINK1 silencing, the activities of complexes I, II+III and IV were all unaffected (data not shown). However, after 12 days of treatment with PINK1 #1 or PINK1 #2 siRNA, the activity of complex IV was significantly inhibited by 50% and 55%, respectively (*P*<0.05; Table 1). The activities of complexes I and II+III were also lower, but did not reach significance.

**Table 1:** ETC activity in SH-SY5Y cells following 12 days of PINK1 silencing.

siRNA	ETC Activity		
	I	II+III	IV
PINK1 #1	14.2±4.1	5.4±1.2	0.068±0.012*
PINK1 #2	13.6±2.6	4.8±0.8	0.059±0.011*
control	20.1±6.4	7.5±1.9	0.132±0.024

SH-SY5Y cells were transfected with PINK1 or scrambled control siRNA for 12 days and the activities of complexes I, II+III and IV were measured. Data are expressed against citrate synthase activity and multiplied by 100. Data are the mean±*s.e.m* (*n* = 5) * *P*<0.05 vs. control siRNA.

### Mitochondrial membrane potential and ATP synthesis

**Figure 3:**
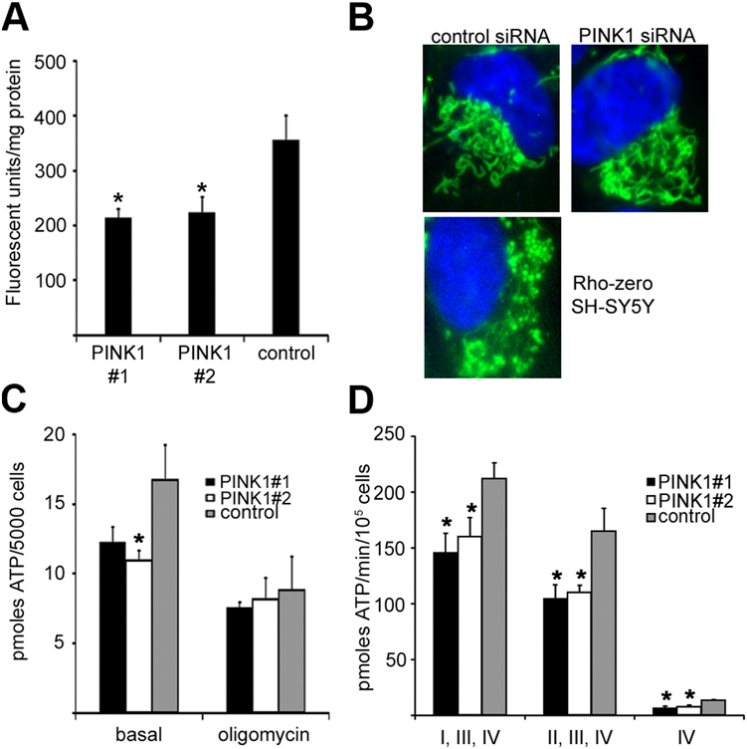
Mitochondrial membrane potential, morphology and ATP synthesis in PINK1-silenced SH-SY5Y cells. Cells were treated with PINK1 or scrambled control siRNA for 12 days. A, mitochondrial membrane potential was assessed by measuring the fluorescence of mitochondrial JC-1 aggregates. Data are expressed against protein and are the mean±*s.e.m.* (*n* = 5). B, live cells were treated with 250 nM MitoTracker Green and 5 µg/ml DAPI, and color images were captured by fluorescent microscopy. Typical mitochondrial networks found in single cells following control or PINK1 siRNA treatment are shown in green. The far right panel shows the disruption of the mitochondrial network in cells pharmacologically depleted of mtDNA (Rho zero cells). The nucleus of each cell is shown in blue. C, steady state cellular ATP levels were measured in siRNA treated cells under basal conditions or following pre-treatment with 1 µg/ml of oligomycin. Data are the mean±*s.e.m*. (*n* = 4). D, ATP synthesis was measured in permeabilized siRNA-treated cells at 37°C using substrates that feed electrons into the electron transport chain at particular points (glutamate+malate (complex I); succinate+rotenone (complex II) or ascorbate+*N*,*N*,*N′*,*N′*-tetramethyl-p-phenylenediamine (complex IV)). Data are the mean±*s.e.m.* (*n*≥5). * *P*<0.05 vs. scrambled control siRNA.

As there was a loss of complex IV activity after 12 days of silencing, mitochondrial membrane potential was assessed at this time point using the JC-1 probe. The fluorescence of mitochondrial JC-1 aggregates was significantly decreased by 40% (PINK1 #1) or 37% (PINK1 #2) in PINK1-silenced cells (*P*<0.05; Figure 3A).

Silencing of PINK1 expression by siRNA in Hela cells has been reported to fragment the mitochondrial network [22]. Therefore, live SH-SY5Y cells treated with siRNA for 12 days were stained with MitoTracker Green to assess the mitochondrial network. No fragmentation of the mitochondrial network was observed following silencing of PINK1 expression under normal culture conditions (Figure 3B). Culturing siRNA treated cells in 0.9 g/l galactose instead of glucose for the last two days of the silencing experiment also had no effect (data not shown). SH-SY5Y cells pharmacologically depleted of mtDNA (Rho zero cells) did exhibit a significant alteration in the mitochondrial network (Figure 3B).

Maintenance of the mitochondrial membrane potential is essential for the generation of ATP by the ETC. Steady state cellular ATP levels were decreased by 34% (*P*<0.05) in PINK1-silenced cells (Figure 3C). The decrease in ATP levels was due to decreased mitochondrial synthesis, rather than increased cellular consumption of ATP (e.g. Na+/K+-dependent ATPases or the proteasome), as pre-treatment of cells with the ATP synthase inhibitor oligomycin abolished the difference in ATP levels between PINK1 and scrambled control siRNA transfected cells. This was further confirmed by measuring the rate of ATP synthesis in digitonin permeabilized cells following 12 days of siRNA treatment. ATP synthesis was significantly inhibited (*P*<0.05; *n*≥5) in PINK1-silenced cells by 31% (PINK1 #1) or 24% (PINK1 #2) using glutamate+malate as substrates (complexes I, III, IV), 36% (PINK1 #1) or 33% (PINK1 #2) with succinate+rotenone (complexes II, III, IV), and 53% (PINK1 #1) or 45% (PINK1 #2) with ascorbate+*N*,*N*,*N′*,*N′*-tetramethyl-p-phenylenediamine (complex IV; Figure 3D).

### MtDNA transcription and translation

Since mtDNA levels were decreased in PINK1-silenced cells, we investigated whether the loss of ETC activity was due to decreased transcription of mtDNA-encoded subunits. Quantitative real-time PCR of the heavy strand transcripts MTCYTB (complex III) and MTCO3 (complex IV), and the light strand transcript MTND6 (complex I) were measured in PINK1-silenced cells. After 12 days, mRNA levels were similar to control siRNA-treated cells (Figure S3A). Western blotting for subunit MTCO2 of complex IV suggested that the significant loss of complex IV activity was not due to decreased translation (Figure S3B), while blue native gels of siRNA-treated cells also indicated that PINK1-silencing did not affect assembly of the complex IV holoenzyme (Figure S3C).

### Oxidative stress and cell death

**Figure 4:**
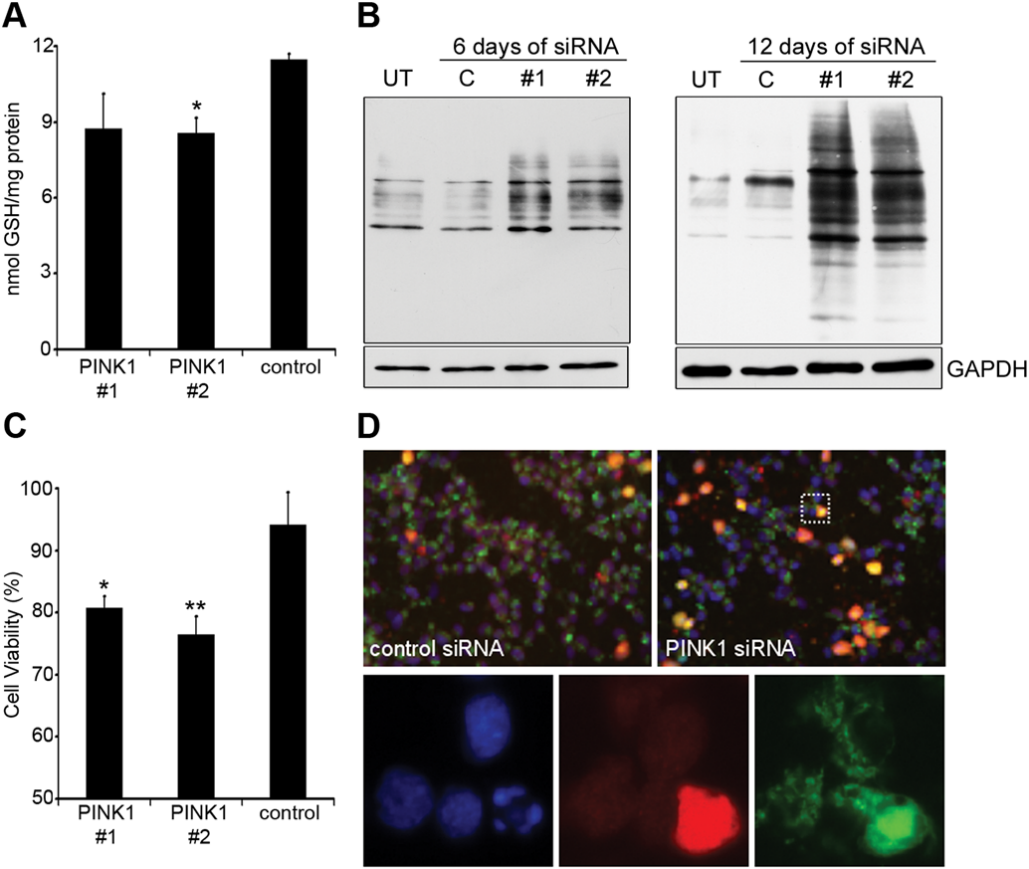
Oxidative stress and cell death in PINK1 silenced cells. SH-SY5Y cells were treated with PINK1 or scrambled control siRNA for 12 days. A, reduced glutathione was measured by reverse-phase high performance liquid chromatography. Data are expressed against protein and are the mean±*s.e.m.* (*n* = 4). B, the carbonylation of protein residues following PINK1 silencing (#1, #2), or in untreated cells (UT), or cells treated with control siRNA (C), was determined by Oxyblot. Cells were treated with siRNA for either 6 or 12 days. Loading of Triton X-100 cell lysates was assessed by re-probing the western blot with GAPDH antibody. C, cell death was measured in siRNA-treated cells by the Cell Titer-Blue assay following treatment with 0.5 mM paraquat for 24 hours. Data are expressed as percentage of fluorescence of vehicle-treated cells grown in sister wells (*n* = 6). D, immunofluorescence of cells following treatment with 0.5 mM paraquat for 24 hours. Cells probed for active caspase-3 (red) and cytochrome-*c* (green). Cell nuclei were counterstained with DAPI (blue). The inset is shown below. * *P*<0.05; ** *P*<0.01 vs. scrambled control siRNA.

Markers of oxidative stress were assessed in PINK1-silenced cells. The levels of reduced glutathione, an antioxidant located in the mitochondria and cytosol, were measured after 6 and 12 days of PINK1 silencing. Reduced glutathione levels were unaffected after 6 days of silencing, a time point at which no inhibition of the ETC was observed. However, reduced glutathione levels were significantly decreased by 25% (*P*<0.05) in PINK1-silenced cells after 12 days (Figure 4A), and was coincident with the decrease in ETC activity. The protein expression of the mitochondrial antioxidant enzyme SOD2 was also investigated in cells following 12 days of PINK1-silencing, but was found to be unaffected (data not shown).

In addition, oxidative stress in SH-SY5Y cells treated with PINK1 siRNA for 6 or 12 days was assessed by measuring the carbonylation of proteins by oxidizing species. A marked increase in carbonylated proteins was observed in PINK1-silenced cells, when compared to untreated or scrambled control siRNA treated cells (Figure 4B). When the density of DNP-positive bands in each lane was measured, and expressed relative to GAPDH, the amount of carbonylated proteins was significantly increased (*P*<0.01; *n* = 4) by four-fold in cells treated with PINK1 siRNA, when compared to control siRNA (PINK1 #1, 6.46±1.14 relative intensity; PINK1 #2, 6.20±0.11; control siRNA, 1.61±0.21). Considerably lower levels of protein carbonyls were detected in cells treated with siRNA for 6 days. An exposure time forty times longer than the 12 day treated samples is shown (Figure 4B). There is no significant increase in the amount of carbonylated proteins in cells treated with PINK1 siRNA for 6 days.

As mentioned earlier, cell viability was unaffected in PINK1-silenced cells grown under basal conditions for 12 days. This was despite the loss of ETC activity and increased oxidative stress that occurs at this time point. However, treatment of these cells with the oxidizing species generator paraquat (0.5 mM) for 24 hours resulted in a significant decrease in cell viability (*P*<0.05), when compared to scrambled control siRNA-transfected cells (Figure 4C). SH-SY5Y cells transfected with PINK1 siRNA for 12 days also showed increased apoptosis following treatment with 0.5 mM paraquat for 24 hours (Figure 4D, upper panels). The number of PINK1-silenced cells immunopositive for active caspase-3 (9.8%) was two-fold higher (*P*<0.05), compared to control siRNA-transfected cells (5.0%). Cells expressing caspase-3 showed a release of cytochrome-*c* from mitochondria and a fragmentation of nuclei (Fig. 4D, lower panels) further confirming their apoptotic state. SH-SY5Y cells transfected with PINK1 siRNA for only 6 days (when ETC and markers of oxidative stress were unaffected) did not show increased sensitivity to paraquat (data not shown).

## Discussion

In this study we report that transfection of dopaminergic SH-SY5Y cells with PINK1 siRNA results in a progressive loss of mitochondrial function. We highlight a putative role for PINK1 in mtDNA synthesis and show for the first time that mitochondrial dysfunction induced by PINK1 directly results in impaired ATP synthesis. Dopaminergic neurons are markedly affected in PD. The loss of PINK1 activity in our dopaminergic cell system has also confirmed previous reports that have shown that mitochondrial dysfunction, and sensitivity to oxidative stress and apoptosis increases over time in mixed brain cell populations [17], [23].

MtDNA has previously been reported to be decreased in *Drosophila* not expressing PINK1 [19]. This coincided with a marked reduction in mitochondrial protein indicating that this decrease was due to loss of mitochondrial number. Our experiments suggest that the depletion of mtDNA in SH-SY5Y cells was due to inhibition of mtDNA synthesis, rather than a loss of mitochondria. It should be noted that mtDNA levels have been reported to be unaffected in the fibroblasts of a single PD patient homozygous for the p.W437X PINK1 nonsense mutation [33].

The mechanism by which PINK1 influences mtDNA is unclear. The decrease in mtDNA levels precedes the loss of ETC complex activity, mitochondrial membrane potential and decreased ATP synthesis. This suggests that the decrease in mtDNA levels is not a consequence of perturbed oxidative phosphorylation. It should also be noted that loss of complex IV activity in patients does not result in decreased mtDNA levels [34].

The regulation of mtDNA levels would also appear not to be mediated by a PINK1-parkin pathway. Despite parkin being implicated in mitochondrial biogenesis [32], over expression of parkin could not reverse mtDNA depletion following silencing of PINK1. The PINK1-parkin pathway has also been shown to affect the morphology and dynamics of the mitochondrial network. Our PINK1-silencing experiments did not result in fragmentation of the mitochondrial network, as was reported in Hela cells [22], and can be also be discounted as a reason for mtDNA depletion. The number of cells required for reliable electron microscopic analysis of mitochondria following siRNA transfection was not experimentally feasible. Therefore it is unclear whether the mitochondria are swollen or have disordered cristae as reported previously [21], [23]. However, it should be noted that a significant reduction in mtDNA levels can also alter mitochondrial morphology (Figure 3B) [35], [36].

Phosphorylation pathways have been found to increase mtDNA levels *in vitro* [37]. A possible protein downstream of PINK1 is DNA polymerase-γ. This protein is involved in the synthesis and repair of mtDNA. Mutations in this gene have been associated with PD [38], [39], although not in all studies [40]. HtrA2 is another protein proposed to be downstream of PINK1 [13]. A recent report has shown a trend for slightly lower mtDNA levels in the brains of HtrA2 knock out mice, although it was not significant [41]. HtrA2 has been proposed to act independently of the PINK1-parkin pathway [42], [43]. Therefore, further investigation of the PINK1-HtrA2 pathway in dopaminergic cells might elucidate the mechanism by which PINK1 affects mtDNA levels.

Despite mtDNA levels being depleted in PINK1-silenced cells, the transcription and translation of mtDNA-encoded subunits were unaffected. Therefore it does not appear that the decrease in mtDNA is the cause of the significant decrease in electron transport chain function observed. Previously it has been reported that the transcription and translation of mtDNA encoded genes is unaffected in cells with 50% mtDNA depletion [44], [45]. Therefore, the levels of mtDNA in SH-SY5Y cells following PINK1 silencing may still be greater than the mitochondrial threshold required for decreased mitochondrial transcription and translation [46].

In agreement with the results of our PINK1 silencing, Piccoli et al. [47] have reported a decrease in complex IV activity in skin fibroblasts derived from a patient homozygous for the PINK1 nonsense mutation p.W437X. A small decrease in complex I activity has also been observed in fibroblasts from PD patients with the homozygous p.G309D PINK1 mutation [48]. The recent finding of impaired mitochondrial respiration in the brains of PINK1 knock out mice [17] is also in agreement with the decreased oxidative phosphorylation we observe following PINK1 silencing. As noted above, loss of PINK1 activity affects oxidative phosphorylation in a variety of cell types. It would be of interest to determine whether the ETC is more effected in dopaminergic neurons versus non-dopaminergic neurons.

Phosphorylation of ETC subunits has been shown to increase ETC activity [37], [49]. Therefore the loss of ETC activity following PINK1 silencing could be due to decreased phosphorylation. Interestingly, oxidative phosphorylation has also been shown to be decreased in the brains of HtrA2 knock out mice [41]. This raises the possibility that a PINK1-HtrA2 pathway might regulate mitochondrial function. Alternatively, loss of ETC function could be a result of oxidative modification (see below) or abnormal mitochondrial morphology.

The silencing of PINK1 in SH-SY5Y cells also resulted in decreased cellular levels of reduced glutathione, a key antioxidant in the brain that has been reported to be lower in the substantia nigra of sporadic PD patients [50]. Total glutathione levels have been shown to be affected in human neurons with PINK1 knock down [23] and human fibroblasts expressing the p.G309D PINK1 mutation [48]. Twelve days of PINK1 silencing also resulted in a significant increase in oxidized protein. Both these markers of oxidative stress coincided with loss of ETC function. It is unclear whether the loss of ETC function is a direct cause of the oxidative stress, or if silencing of PINK1 increases oxidative stress via another pathway, which in turn progressively inhibits the ETC and other cellular components by oxidative modification. Increasing damage to the ETC components may explain why it takes twelve days for loss of ETC activity to be observed. It should be noted that loss of mitochondrial respiration in the brains of PINK1 knock out mice increases with age, as does sensitivity to hydrogen peroxide [17], while oxidative modification of complex I has also been observed in PD brains [51].

The decrease in mitochondrial membrane potential following PINK1 silencing reported here has also been reported in other human cell models [22], [23]. The mitochondrial membrane potential is generated by the ETC and is essential for the synthesis of ATP by mitochondria. Therefore it has been suggested that the loss of mitochondrial membrane potential observed following PINK1 silencing will have a significant effect on ATP production, and thus cellular function and survival. Indeed, a reduction in ATP levels has been observed in PINK1 fly knock out models that also exhibit neuronal loss [19], [20]. For the first time, we have demonstrated directly that mitochondrial ATP synthesis is inhibited in cells depleted of PINK1, and that this event is concomitant with both a decrease in mitochondrial membrane potential and inhibition of complex IV of the ETC.

Although mitochondrial dysfunction and oxidative stress occur after 12 days of PINK1-silencing, there was no effect on cell viability under basal conditions. However, when these cells were treated with paraquat, they exhibited increased cell death when compared to siRNA control cells. A combination of both decreased ATP production and intracellular oxidative stress most likely contributes to the increased susceptibility to oxidizing species. This is supported by the observation that after 6 days of PINK1 silencing, when no ETC dysfunction or oxidative stress was observed, there was no increase in sensitivity to paraquat. This increase in mitochondrial dysfunction and susceptibility to apoptosis or oxidative stress over time has also been reported in brain cells with perturbed PINK1 activity [17], [23].

In summary, loss of PINK1 activity results in a progressive loss of mitochondrial function. PINK1 silencing results in decreased mtDNA levels, impaired oxidative phosphorylation and oxidative stress. The decreases in mtDNA and ETC activity do not appear to be directly related. The mechanisms responsible for these two events are unclear. The loss of mtDNA precedes marked levels of oxidative stress, and can probably be discounted as a cause. Conversely, impaired oxidative phosphorylation and increased oxidative stress coincide. Further experiments are required to ascertain whether the oxidative stress is a cause or consequence of ETC inhibition, or an unrelated event. The effects we report following loss of PINK1 expression are common to events recognized in sporadic form of PD. Therefore, elucidation of PINK1 function will probably give a better understanding of the pathogenic pathways leading to both forms of PD.

## Supporting Information

Figure S1 Endogenous and recombinant PINK1 protein expression in SH-SY5Y cells. SH-SY5Y cells were transfected with empty vector (pCMV6Neo) or vector containing PINK1 (pCMV6Neo-PINK1). Full-length (60 kDa) and processed (∼55 kDa) recombinant PINK1 were detected by western blotting and are indicated by an arrow and arrowhead, respectively. Long exposure yielded a faint band corresponding to full-length endogenous PINK1 in SH-SY5Y transfected with empty vector. Asterisk denotes non-specific band co-migrating with processed PINK1. Equal protein loading was verified by an antibody specific for the SDH subunit of complex II.
(0.29 MB TIF)

Figure S2 MtDNA levels in SH-SY5Y cells over-expressing parkin. SH-SY5Y cells or SH-SY5Y cells over-expressing parkin were treated with PINK1 siRNA or control siRNA for 6 days, and mtDNA levels measured by quantitative real-time PCR. MtDNA levels were expressed relative to the single copy nuclear gene TK2 and are expressed as the mean±SEM (n = 4). * P<0.05 vs. SH-SY5Y treated with scrambled control siRNA; # P<0.05 vs. SH-SY5Y cells over-expressing parkin treated with scrambled control siRNA.
(0.05 MB TIF)

Figure S3 Transcription and translation of mtDNA genes in PINK1-silenced SH-SY5Y cells. (A) SH-SY5Y cells were treated with PINK1 or scrambled control siRNA for 12 days and total cellular RNA extracted. Transcript levels from the mtDNA genes MTND6, MTCYTB and MTCO3 were measured by qPCR. mRNA levels were expressed relative to GAPDH mRNA and are expressed as the mean±SEM (n = 3). Protein lysates of SH-SY5Y cells treated with PINK1 or control siRNA for 12 days were prepared and separated by SDS-denaturing (B) or blue native gels (C). Expression of subunit II of complex IV (MTCO2) and assembled complex IV (CXIV) were then determined by western blotting. Equal protein loading was verified on each blot with antibodies specific for porin or complex II (CXII).
(0.27 MB TIF)

Table S1 Primer sequences for quantitative real time PCR
(0.03 MB DOC)

Method S1 Blue native gels. Method for blue native electrophoresis that is shown in Figure S3
(0.02 MB DOC)
