# Supplementary figures and images for: Silencing of PINK1 Expression Affects Mitochondrial DNA and Oxidative Phosphorylation in DOPAMINERGIC Cells
Source: PLoS One. 2009 Mar 9;4(3):e4756. doi: 10.1371/journal.pone.0004756 (PMC2649444; doi:10.1371/journal.pone.0004756)

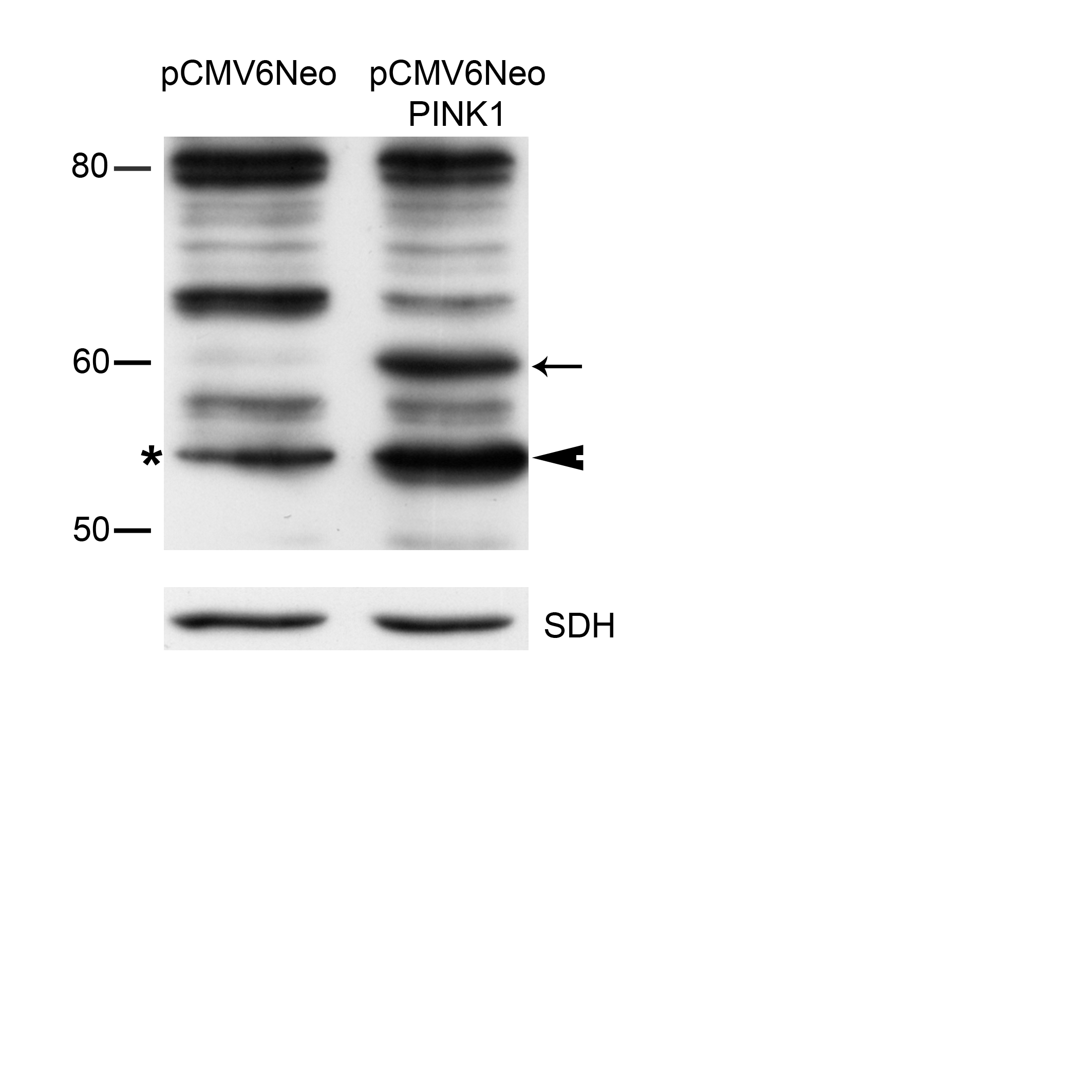

Supplement: Figure S1 — Endogenous and recombinant PINK1 protein expression in SH-SY5Y cells. SH-SY5Y cells were transfected with empty vector (pCMV6Neo) or vector containing PINK1 (pCMV6Neo-PINK1). Full-length (60 kDa) and processed (∼55 kDa) recombinant PINK1 were detected by western blotting and are indicated by an arrow and arrowhead, respectively. Long exposure yielded a faint band corresponding to full-length endogenous PINK1 in SH-SY5Y transfected with empty vector. Asterisk denotes non-specific band co-migrating with processed PINK1. Equal protein loading was verified by an antibody specific for the SDH subunit of complex II. (0.29 MB TIF) [file pone.0004756.s001.tif]

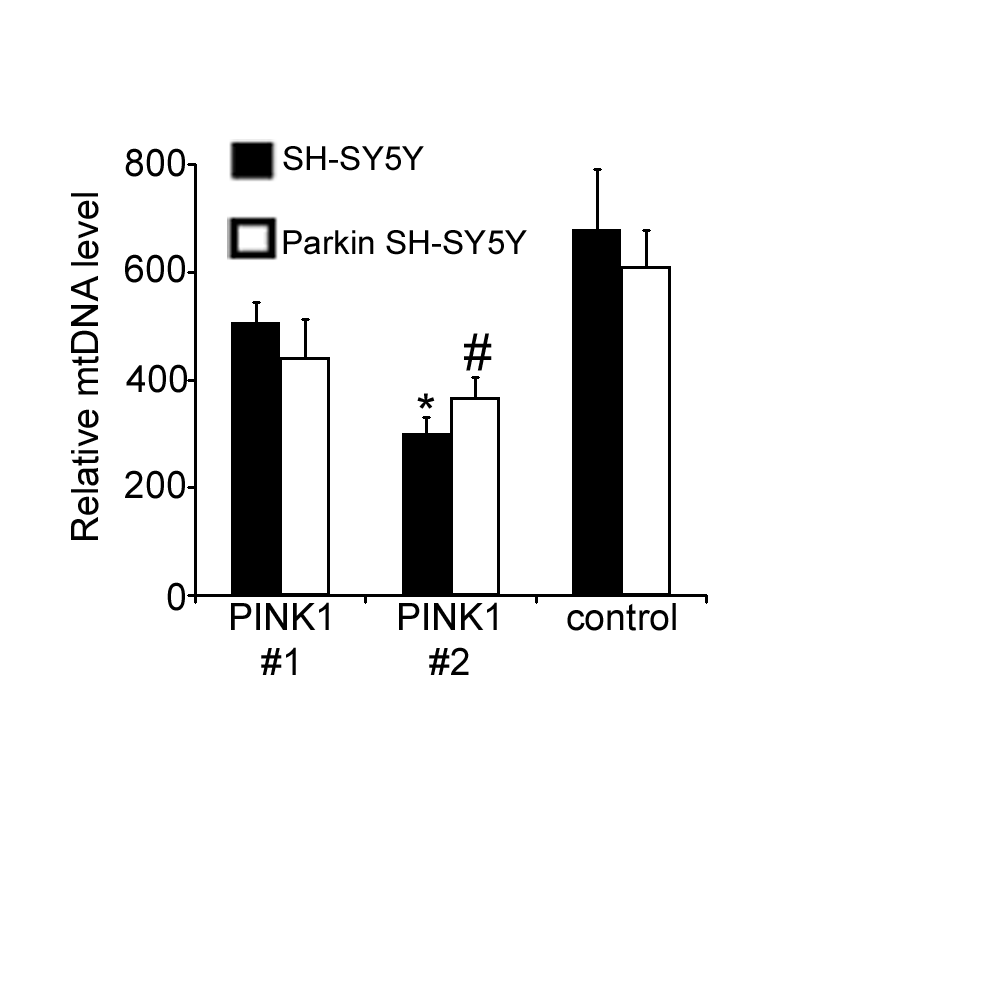

Supplement: Figure S2 — MtDNA levels in SH-SY5Y cells over-expressing parkin. SH-SY5Y cells or SH-SY5Y cells over-expressing parkin were treated with PINK1 siRNA or control siRNA for 6 days, and mtDNA levels measured by quantitative real-time PCR. MtDNA levels were expressed relative to the single copy nuclear gene TK2 and are expressed as the mean±SEM (n = 4). * P<0.05 vs. SH-SY5Y treated with scrambled control siRNA; # P<0.05 vs. SH-SY5Y cells over-expressing parkin treated with scrambled control siRNA. (0.05 MB TIF) [file pone.0004756.s002.tif]

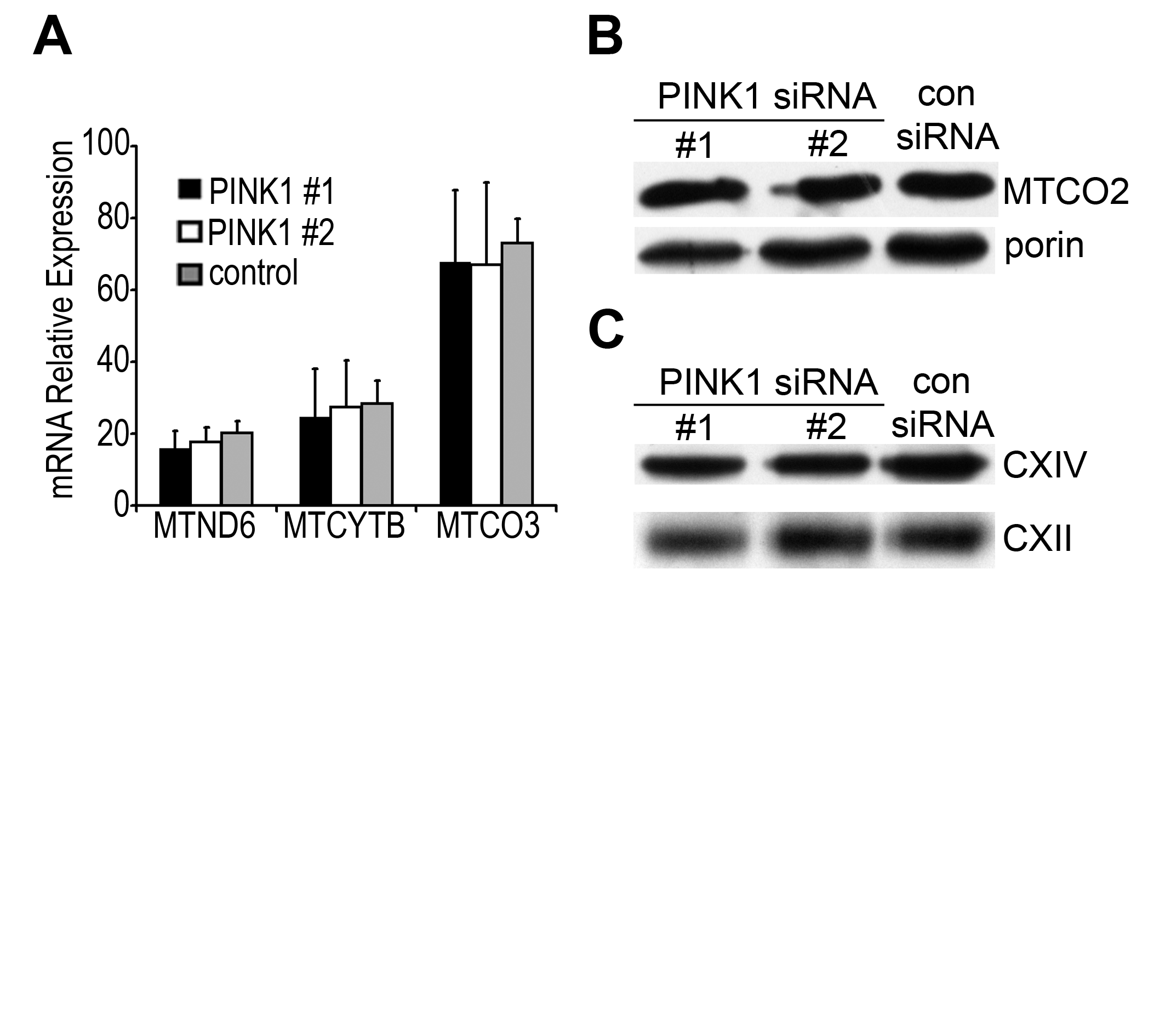

Supplement: Figure S3 — Transcription and translation of mtDNA genes in PINK1-silenced SH-SY5Y cells. (A) SH-SY5Y cells were treated with PINK1 or scrambled control siRNA for 12 days and total cellular RNA extracted. Transcript levels from the mtDNA genes MTND6, MTCYTB and MTCO3 were measured by qPCR. mRNA levels were expressed relative to GAPDH mRNA and are expressed as the mean±SEM (n = 3). Protein lysates of SH-SY5Y cells treated with PINK1 or control siRNA for 12 days were prepared and separated by SDS-denaturing (B) or blue native gels (C). Expression of subunit II of complex IV (MTCO2) and assembled complex IV (CXIV) were then determined by western blotting. Equal protein loading was verified on each blot with antibodies specific for porin or complex II (CXII). (0.27 MB TIF) [file pone.0004756.s003.tif]
