# Supplementary material for: Silencing of PINK1 Expression Affects Mitochondrial DNA and Oxidative Phosphorylation in DOPAMINERGIC Cells
Source: PLoS One. 2009 Mar 9;4(3):e4756. doi: 10.1371/journal.pone.0004756 (PMC2649444; doi:10.1371/journal.pone.0004756)
Supplement: Table S1 — Primer sequences for quantitative real time PCR (0.03 MB DOC) [file pone.0004756.s004.doc]

### TABLE S1. Primer sequences for quantitative real time PCR

| **Target** | **Sequence** | **Annealing Temp (°C)** |
| --- | --- | --- |
| MTCYTB | 5’-GAC CTC CCC ACC CCA TCC A-3’  5’-AAA GGC GGT TGA GGC GTC TG-3’ | 60 |
| MTCO3 | 5’-GCC CTC TCA GCC CTC CTA ATG-3’  5’-GTG GCC TTG GTA TGT GCT TTC TCG-3’ | 60 |
| D-loop | 5’-CAT CTG GTT CCT ACT TCA GGG-3’  5’-TGA GTG GTT AAT AGG GTG ATA GA-3’ | 60 |
| GAPDH | 5’-GAA GGT GAA GGT CGG AGT-3’  5’-GAA GAT GGT GAT GGG ATT TC-3’ | 56 |
| MTND6 | 5’-TGG ATA TAC TAC AGC GAT GGC-3’  5’-AAC CAC CAC CCC ATC ATA C-3’ | 56 |
| PINK1 | 5’-GGA CGC TGT TCC TCG TTA-3’  5’-ATC TGC GAT CAC CAG CCA-3’ | 56 |
| *TK2* | 5’-TCC TGC AGA TGC CAC TTT GA-3’  5’-CCC CAA GTC TGA AGA AAA CG-3’ | 60 |
