# Supplementary material for: Silencing of PINK1 Expression Affects Mitochondrial DNA and Oxidative Phosphorylation in DOPAMINERGIC Cells
Source: PLoS One. 2009 Mar 9;4(3):e4756. doi: 10.1371/journal.pone.0004756 (PMC2649444; doi:10.1371/journal.pone.0004756)
Supplement: Method S1 — Blue native gels. Method for blue native electrophoresis that is shown in Figure S3 (0.02 MB DOC) [file pone.0004756.s005.doc]

**Supplementary Method**

Blue Native Gels

n-dodecyl-β-D-maltoside lysates were prepared as for SDS-PAGE and mixed 1:1 with protein solubilising solution (1M 6-aminocaproic acid, 50 mM bistris, 1% Servablue G) and resolved by 8-16% blue native gels (Schägger, 1995). After transfer to Hybond P, blots were probed for complex IV holoenezyme using a monoclonal antibody against MTCO1 (clone 1D6, Mitosciences). Bands corresponding to fully assembled cytochrome oxidase were confirmed with purified bovine complex IV. Protein loading was assessed by reprobing the blot with a complex II specific antibody.

### Schägger H. (1995) Quantification of oxidative phosphorylation enzymes after blue native electrophoresis and two-dimensional resolution: normal complex I protein amounts in Parkinson's disease conflict with reduced catalytic activities. Electrophoresis 16: 763-770.
